# Effectiveness of Lidocaine with Epinephrine Irrigation in Reducing Acute Pain from Surgical Removal of Mesioangular-Impacted Third Molars

**DOI:** 10.3390/dj12120412

**Published:** 2024-12-17

**Authors:** Vuttinun Chatupos, Molee Apiphathanamontri, Sumatee Yuthavong, Piyanart Chatiketu, Nuntouchaporn Hutachok, Somdet Srichairatanakool

**Affiliations:** 1 Department of Oral and Maxillofacial Surgery, Faculty of Dentistry, Chiang Mai University, Chiang Mai 50200, Thailand; vuttinunch@yahoo.co.th (V.C.); ssomdet@hotmail.com (M.A.); Nick_Sumatee@hotmail.com (S.Y.); 2 Residency Training Program in Oral and Maxillofacial Surgery, Faculty of Dentistry, Chiang Mai University, Chiang Mai 50200, Thailand; 3 Department of Family and Community Dentistry, Faculty of Dentistry, Chiang Mai University, Chiang Mai 50200, Thailand; pichatiketu@gmail.com; 4 Department of Biochemistry, Faculty of Medicine, Chiang Mai University, Chiang Mai 50200, Thailand; thenuntouch@gmail.com

**Keywords:** anesthetic, lidocaine, nerve block, pain, third molar, VAS

## Abstract

Background: Anesthetic irrigation is an effective treatment for postoperative pain suppression in patients after molar extraction, but exerts a short period of extraction. The study aimed to evaluate the effect of lidocaine with epinephrine irrigation on acute pain relief in healthy volunteers with inferior alveolar nerve block (IANB) injection for the surgical removal of mesioangular (MA)-impacted third molars. Methods: A total of 28 patients (56 samples) with bilateral MA-impacted third molars were recruited. This study was a split-mouth, double-blind, randomized clinical trial. Surgical procedures were conducted over two separate appointments. Each patient randomly received 2% lidocaine with 1:100,000 epinephrine for the irrigation of the extraction site after surgery on one side of the mouth and normal saline solution on the other side. The postoperative visual analog scale (VAS) as a pain indicator was recorded and statistically analyzed for both treatments. Results: The VAS levels at 3 and 4 h after surgery in the lidocaine group were significantly lower than those of the normal saline group. Increases in pain scores were recorded five hours after surgery in both groups. No complications were recorded during this study. Conclusions: Continuous local anesthetic irrigation appears to be effective in reducing acute postoperative pain in patients with IANB for MA-impacted third molar surgery. Clinical Trial Registry: Reference number ISRCTN13866362, Date: 1 October 2024.

## 1. Introduction

An impacted mandibular molar is a tooth that is partially or completely unerupted against another tooth and varies with race, age, and other factors (e.g., epigenetics, gender, decreased mesiodistal crown diameter, and nature of diet) [1]. The impacted molars are classified as vertical, mesioabgular, horizontal, distoangular, buccoangular, linguoangular, inverted, and unusual types. Patients with impacted molar teeth may or may not show common symptoms such as pain, dry socket, dental caries, sensory nerve damage, gingivitis, hemorrhage, and oral and throat infections. A previous study has revealed Thai patients aged 21–30 years had maxillary and mandibular third molar impacted teeth (wisdom teeth) that were classified into mesioangular (MA), horizontal, vertical, distoangular, and linguoversion or buccoversion types, in which the MA type is the most common [2]. In comparison, MS and distoangular impactions were found to be the most common types (53.4% and 22.1%, respectively) among Nigerian patients [3]. In addition, females have a higher incidence of impacted mandibular third molars than males [1]. Regarding adverse effects, surgical removal of MA-impacted mandibular third molars can lead to various complications and pathologies such as paresthesia, abnormal bleeding, alveolar osteitis (dry socket), trismus, swelling, and pain.

Postoperative pain is a common side effect among patients undergoing impacted third molar surgery [4]. Considering ways for postoperative anesthesia, primary interventions with laser radiation, corticosteroids (e.g., dexamethasone), hydroxybutyl-chitosan hydrogel dressing, and combined nimesulide or nimesulide with thiocolchicoside treatment are required to control the pain, swelling, and trismus [5,6,7,8]. Accordingly, local anesthetics such as etidocaine, lidocaine, lignocaine, bupivacaine, levobupivacaine, clonidine, ropivacaine, and mepivacaine are used for postoperative pain control in oral surgery, but adverse local and systemic effects, including prolonged duration of actions, metabolic acidosis, and greater hemorrhage, are observed. In addition, anesthetic irrigation has been considered an effective treatment for postoperative pain suppression in patients after undergoing a cesarean section, combined heat and kidney transplantation, and breast augmentation [9,10,11]. Even with a short duration of action, it has been found to effectively suppress postoperative pain for a few hours. Inferior alveolar nerve block (IANB) is a standard technique used in administering local anesthetic to stabilize surrounding tissues at the surgical site [12]. After the anesthetic effect has diminished, the patient can experience a range of physical and mental discomforts. Epinephrine (or adrenaline) is a vasoconstrictor associated with local anesthesia most commonly used in dentistry treatment. In administration, epinephrine is added at specific dilutions (1:80,000–1:200,000) to local anesthetics and subcutaneously injected into patients to produce IANB, and provide excellent anesthesia of the maxillary tooth and hemostasis [13]. It is known that combined treatment of lidocaine and epinephrine showed a fast onset with no delayed effects on wound healing or wound tensile strength [14]. In addition, lidocaine has a shorter duration of action when compared with bupivacaine and has rarely been associated with any allergic case reports [15].

According to previously published literature, anesthetic irrigation using bupivacaine has received positive feedback for postoperative pain control in the surgical removal of the lower third molar [16]. Importantly, epinephrine present in the injection and irrigating solution may contribute to the onset of ventricular tachycardia and potentially cause pre-existing cardiovascular complications during surgery [17]. We expect that the commercially available local anesthetic, lidocaine, combined with epinephrine may produce a promising effect on postoperative outcomes among patients undergoing tooth extraction. In this study, we endeavored to evaluate the effect of lidocaine combined with epinephrine irrigation on acute postoperative pain suppression in patients undergoing the surgical removal of their impacted third molars with mesioangular angulation. 

## 2. Materials and Methods

### 2.1. Drugs

Lignospan Special (2% lidocaine hydrochloride with 1:80,000 epinephrine) (2.2 mL/cartridge) anesthetic for injection and dental use was purchased from Septodont Company (limited), Maidstone, Kent, UK. Amoxicillin capsules (GPO MOX, 500 mg size) were purchased from the Government Pharmaceutical Organization, Bangkok, Thailand. Paracetamol (Beramol, 500 mg tablets) was purchased from B.M. PHARMACY Company (limited), Bangkok, Thailand. Sterile 0.85% sodium chloride or normal saline solution (NSS) was obtained from a drug preparation unit at the Faculty of Dentistry, Chiang Mai University. 

### 2.2. Ethics

This study received approval from the Human Experimentation Committee Research Institute for Health Sciences and was signed by Professor Dr. Anak Iamaroon, D.D.S., M.S., Ph.D., a Chairman of the Committee, Faculty of Dentistry, Chiang Mai University, Chiang Mai 50200, Thailand (Certificate Number: 52/2014, Date: 3 December 2014). Adherence to ethical guidelines was of prime importance throughout the study process. All patients were fully informed about the particulars of the study and willingly provided their signatures on the consent forms before any study procedures were performed. This study followed the guidelines of the Helsinki Declaration 2008, revised in 2013: Ethical Principles for Medical Research Involving Human Subjects. Subjects’ rights have been protected by an appropriate Institutional Review Board, and written informed consent was granted by all subjects.

### 2.3. Clinical Trial Registry

The clinical study protocol was registered, reviewed, and approved by the International Standard Randomized Controlled Trial Number (ISRCTN) Committee of the BioMed Central (Reference number: ISRCTN3866362, Date: 1 October 2024), as can be viewed at the following link: https://www:isrctn.com/ISRCTN13866362: Lidocaine and epinephrine mixture relieves pain from impacted molar surgery (accessed on 15 October 2024). This study was conducted in accordance with the reporting guidelines of the Consolidated Standards of Reporting Trials (CONSORT) 2010 [18].

### 2.4. Patients’ History

The medical history of the participants was recorded in an outpatient department (OPD) card at OPD 4 (Oral and Maxillofacial Surgery Clinic) and obtained through an electronic form used in the Dentistry Faculty. In the card, the information included age, gender, the use of medication, clinical symptoms, the severity of illness, and the presence of other pre-existing medical conditions.

### 2.5. Patient Preparation

Patients who had registered for the surgical removal of their lower third molars at the Oral and Maxillofacial Surgery Clinic, the Faculty of Dentistry, Chiang Mai University, were recruited from October 2015 to February 2016 for this study. The inclusion criteria were as follows: patients of ages between 18 to 25 years of all genders, bilateral MA-impacted lower third molars (George Winter classification) [19], no systemic diseases (American Society of Anesthesiologists Class 1) [20], no history of allergic reactions to the local anesthetic, the lidocaine–epinephrine mixture, and no history of allergic reactions to amoxicillin or paracetamol. Patients experiencing signs and symptoms of oral inflammation, including acute inflammation of the lower third molar or pericoronitis, for at least one month before applying to the program were excluded. This condition was considered since these variabilities could affect the efficiency of anesthetic irrigation and the assessment of any postoperative complications. The operational procedure and any informative information were explained to the patients before they were asked to sign the consent form. Patients were notified that they could withdraw from the study at any time.

### 2.6. Sample Size Calculation

The sample size was calculated according to the data obtained from a study conducted by Rauten et al. [21] in combination with the data obtained from a study conducted by Yamano et al. [22] using STATA^®^ version 16.0 software (StataCorp, LLC, College Station, TX, USA). Initially, the total sample size was 24. Nevertheless, the researchers decided to increase the sample size to a total of 28 individuals, who were then divided into two treatment groups (28 samples each), by including additional participants.

### 2.7. Study Design and Clinical Evaluation

According to the inclusion criteria, a total of 28 patients were enrolled in this study. By using the block randomization method, the participants were categorized into two treatment groups—group 1: third molar surgery (28 samples) with normal saline solution (NSS) irrigation (control) and group 2: third molar surgery (28 samples) with NSS plus lidocaine irrigation (test). The study design implemented a split-mouth, double-blind, randomized clinical trial, which followed the Consolidated Standards of Reporting Trials (CONSORT) 2010 guidelines. All patients attended the surgical procedure over two sessions that were two weeks apart. Patients were interviewed about their health history and the medications they had been prescribed for one month before attending the surgery. The blood pressure of each patient was recorded 30 min before the surgery.

### 2.8. Study Variables 

The dependent variables assessed in the study were pain intensity (in the immediate postoperative period, and at the third, fourth, and fifth hours). The independent variables were the volume of local anesthetic, duration of the surgical procedure, and quantity of analgesic medications ingested.

### 2.9. Subject Allocation and Randomization 

The random allocation of the patients into two groups was conducted using the Microsoft Excel software version 2410 (Microsoft Office Professional Plus 2019, Microsoft Corporation, Redmond, WA, USA) shared license by Chiang Mai University, Thailand. Accordingly, the randomization of 28 subjects proceeded by allocating random permutations of two treatments (1 = NSS and 2 = lidocaine with epinephrine) within each block size (randomly between 4 and 8). The randomization results were placed in sealed envelopes and prepared by a person external to the research team. At the time of irrigation, only the person applying the therapy opened the envelope and had access to information about the allocation of the irrigating solution. 

### 2.10. Surgical Intervention

All surgical procedures were performed by one oral and maxillofacial surgeon, and all procedures were monitored by experienced health care staff. Before suturing each wound, all secretions were completely discarded from the surgical area, and the tooth socket was irrigated with either normal saline solution (NSS) or 2% lidocaine with 1:100,000 epinephrine twice for a period of five minutes. Each disposal syringe contained 3.6 mL of solution, while patients received 7.2 mL of solution in total. After the surgical procedure was completed, patients received amoxicillin at a dose of 500 mg. Specifically, patients were given twelve tablets of amoxicillin and were instructed to take three tablets after each meal for four days straight to avoid oral inflammation. Paracetamol at a dose of 500 mg was also made available to all patients in 10-tablet prescriptions, and they were instructed to take one tablet every six hours if needed to relieve their pain. Postoperative variables, including postoperative pain and the amount of paracetamol consumption, were monitored for 3, 4, and 5 h intervals over seven days, respectively, and the postoperative pain was evaluated using a Visual Analog Scale (VAS).

### 2.11. VAS Assessment 

VAS is a unidimensional tool used in epidemiologic and clinical research to measure the intensity or frequency of various symptoms such as pain from none (0) to extreme amounts, 0 being for no pain and 10 for maximum pain [23]. Firstly, the examiner was calibrated to evaluate pain intensity by using the VAS test, performed by two independent examiners with experience in clinical dentistry. In the assessment, subjects marked on the ruler line the point that they felt, representing their perception of their current pain, and the examiner recorded their pain levels at time intervals covering 2–8 h post-operation, according to the frequency and severity of the pain. Accordingly, the VAS score was determined by measuring the distances from the left-hand end of the line (0 cm) to the point that the patient marked. Following cut points on the pain, VAS values have been recommended: no pain (0–0.4 cm), mild pain (0.5–4.4 cm), moderate pain (4.5–7.4 cm), and severe pain (7.5–10 cm); nonetheless, normative values are not available.

### 2.12. Postoperative Medication

Subjects were prescribed to take one ampicillin capsule after meals and before bedtime with/without one paracetamol tablet every 6 h. If taken, the number and duration of the paracetamol were recorded. Three weeks later, another side of the molar extraction was scheduled and performed as previously described.

### 2.13. Statistical Analysis

**Figure 1:**
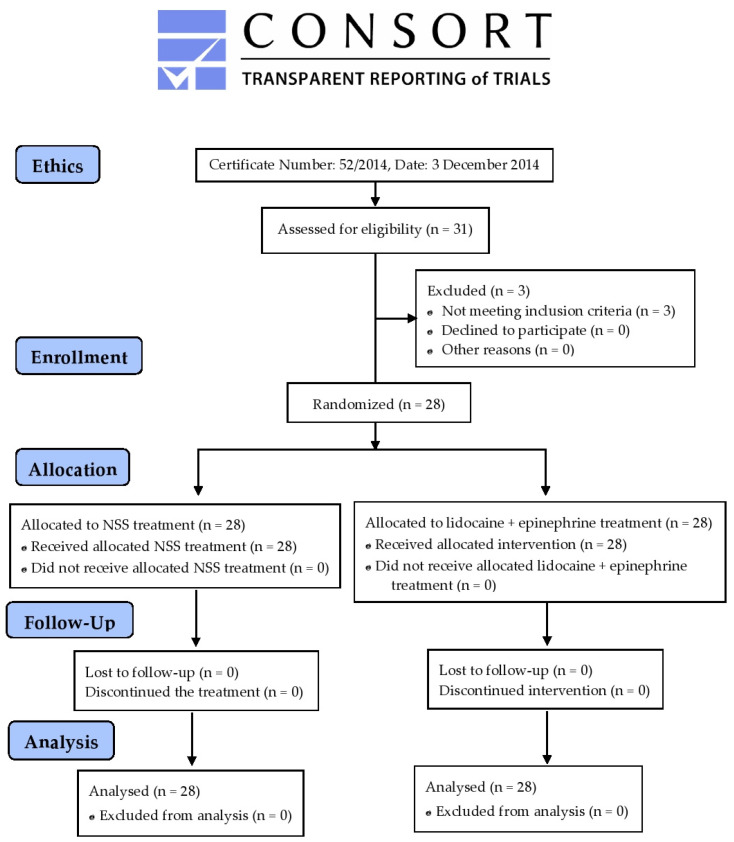
A flowchart of inclusion, randomization, study design, and treatment of patients (*n* = 28) with bilateral MA-impacted third molars (28 samples each).

Data obtained were tabulated into an electronic database in the Excel software version 2410 (Microsoft Office Professional Plus 2019). A descriptive analysis was performed using the Statistical Package for the Social Sciences (SPSS) Program (IBM SPSS Software version 22, IBM Corporation, Armonk, NY, USA, shared license by Chiang Mai University, Thailand). Quantitative variable data were expressed as mean ± standard deviation (SD) values. The Shapiro–Wilk test was used for the data distribution (normal or non-normal). Results for the duration of the surgical procedure, pain score, volume of local anesthetic, and quantity of analgesic medications taken were analyzed using the Paired *t*-test for parametric data and the Wilcoxon signed rank test for non-parametric data to compare between the NSS and lidocaine with epinephrine groups. Comparisons between time periods in the same group were performed using a one-way analysis of variance (ANOVA), followed by Fisher’s Least Significant Difference (LSD) post hoc test. The LSD is based on the smallest difference among the two means, which is considered to be significant at a particular level of significance. Accordingly, a *p*-value < 0.05 was considered statistically significant. The consolidated standards of reporting trial (CONSORT) flow diagram of this study is shown in Figure 1.

## 3. Results

### 3.1. Participant Information

A total of 31 patients were enrolled in this study; however, 3 patients did not meet the inclusion criteria and were excluded. Accordingly, a total of 28 patients, including 13 males (46.43%) and 15 females (53.57%), with a mean age of 21.75 ± 1.65 years and a range of 18.3–25.0 years continued to participate in this study (Table S1). The average volume of NSS and lidocaine with epinephrine used to cleanse the surgical area and the duration of each surgery were similar among the control and treatment groups (Table 1). No postoperative complications were reported during the surgical procedures.

### 3.2. VAS Scores

Patients who received the NSS irrigation exhibited no significant differences in their VAS scores five hours after the surgery. On the contrary, patients who received lidocaine with epinephrine irrigation showed lower VAS scores than the NSS irrigation group, and the values showed a gradual increase over time (Table 2). 

The highest VAS score was reported for the period between three to four hours after surgery in the control group (48.67 ± 21.43) and four to five hours in the treatment group (39.74 ± 19.73). Thus, VAS scores were considered significantly different between the two groups at three and four hours after surgery.

### 3.3. Analgesic Effect Associated with Paracetamol

The average amount of paracetamol consumption in the control and treatment groups were decreased gradually, as was proportional to the postoperative day (Table 3). On days 1 to 5, paracetamol intakes were significantly higher in the normal saline group when compared with the lidocaine with epinephrine group. There were no significant changes in paracetamol usage among either group at the late postoperative phase.

## 4. Discussion

This study aims to evaluate the effect of lidocaine with the vasoconstrictor, epinephrine, on acute postoperative pain suppression in patients undergoing surgical removal of the impacted third molar. For each patient, the surgical site on one side of the mouth was irrigated with sterile normal saline during the first surgery, while the other side was irrigated with a local anesthetic, lidocaine combined with epinephrine, during the second surgery. Our results indicate that the irrigation of lidocaine with epinephrine did not influence the volume used to cleanse the surgical area and the molar surgery duration, whereas neither side effects nor complications were found during or after surgery. Interestingly, lidocaine with epinephrine suppressed acute pain over periods of 3, 4, and 5 h after surgery, in which the significance was found at the time points of 3 and 4 h. The prolongation of postoperative periods promoted significant increases in the VAS scores in patients administered with lidocaine with epinephrine irrigation. The VAS scores were significantly lower in the lidocaine with epinephrine group when compared with the normal saline group. We assume that the irrigation of the anesthetic directly to the tooth socket may have promoted the direct contact of the anesthetic to the inferior alveolar nerve below the wound margin and, consequently, prolonged the duration of the anesthetic effect. Rodrigues et al. reported that lidocaine played a potential role in nociceptive fiber synapsis suppression, which could have resulted in the suppression of the neurotransmitter, substance P [24]. This substance is a critical neurotransmitter that is administered in many important situations such as mast cell-promoted cytokine release [25]. At the point of the depletion of the neurotransmitter, the inflammatory cytokines were suppressed, which led to pain reduction. Epinephrine injection prior to surgery also helped to relieve pain by influencing vasoconstriction of the blood vessels located in and around the tooth socket, which slowed the absorption of the anesthetic into the blood, prolonged the action of the anesthetic, and inhibited local bleeding. Few studies have reported on the use of this lidocaine–epinephrine mixture in postoperative irrigation for impacted third molar surgery.

We believe that acute oral infection is a critical complication that can occur from lidocaine irrigation. However, no incidences of infection were reported in this study. According to a study conducted by Eshghpour et al., the incidence rate of acute infection at the dry socket occurred twice as often when more than two cartridges of the anesthetic agent were applied [26]. This complication may have been influenced by the vasoconstrictor agent composed of the anesthetic. Vasoconstrictor agent overdose can promote serious complications such as peripheral vascular disease, hypoxemia, delayed wound healing, and suppressed fibrin degradation [27]. According to the outcomes of previous studies, lidocaine is able to induce mitochondria membrane degradation [28], while incidences of chondrotoxicity have also been reported [29]. Lidocaine has been reported to suppress collagen generation, but it did not affect wound strength [14,30]. Lidocaine at concentrations of 1% and 2% increased the wound healing rate, while epithelial regeneration has also been observed in rats [31]. Notably, lidocaine irrigation did not promote any delay in wound healing in this study. In limitations, this is a small study that would now benefit from larger numbers. Other possible relations and most effective compositions of lidocaine with epinephrine will be explored to minimize the postoperative sequelae associated with mandibular third molar surgery. The pain evaluation method, simply using VAS, is dependent on the perception and pain intensity reported by the subjects, which may not reliably describe or inform the biological effects on the molar extraction in relation to its analgesics. Since the bilateral MA-impacted third molars were separated on different days (two weeks apart), it is impossible to distinguish which side had effects of pain perception or reduced mouth opening. Moreover, additional in vitro, artificial intelligence-assisted, and clinical methods need to be explored for more precise, susceptible, feasible, and reliable assessments in pre- and postoperative dental cares, particularly following the removal of mandibular third molars. For instance, trapezoidal, marginal, papilla detachment, and papilla decapitation flaps techniques may be applied to evaluate wound healing after lower molar surgery, as previously reported in periodontal healing [32]. Additionally, platelet-rich fibrin comprising platelets, cytokines, and growth factors will be necessarily used for wound healing and to prevent postoperative complications [33]. Furthermore, our working group is presently conducting other ongoing projects in this same line of research: for example, reliable biomarkers for pain assessment compared with VAS and analgesic and anti-inflammatory effects of anthocyanins-rich extract from kale (*Brassica oleracea*). 

## 5. Conclusions

Lidocaine can be used safely and effectively in routine dental surgery in healthy and impacted third-molar surgery patients. Herein, local anesthetic irrigation by 2% lidocaine with epinephrine (a dilution of 1:100,000), continuously for five minutes, exhibited effectiveness in reducing acute postoperative pain at 3, 4, and 5 h intervals in patients undergoing mesioangular-impacted third molar surgery. In future studies, higher evidenced clinical trials are hoped to indicate more parameters and biomarkers that can influence the pain intensity and complications in postoperative removal of lower third molars. In alternative medication, potential plant-derived extracts showing analgesic and anti-inflammatory properties need to be investigated in controls of the pain and complications.

## Figures and Tables

**Table 1:** Information was obtained from the NSS and lidocaine with epinephrine treatment groups during the operation. Data are expressed as mean ± SD values (*n* = 28). A paired *t*-test was used to determine a significant difference, where ^NS^ = nonsignificant when compared with a placebo (NSS) group.

Rinsing Solution	Factors	
	Volume of Local Anesthetic (mL)	Duration of Surgery (min)
NSS	2.03 ± 0.38	43.21 ± 7.34
Lidocaine with epinephrine	1.96 ± 0.33 ^NS^	41.87 ± 6.85 ^NS^

**Table 2:** Comparison of VAS scores in patients who received different irrigation preparations at each postoperative time period. Data are expressed as mean ± SD values (*n* = 28). * Paired *t*-test and ^#^ ANOVA test were used to determine significant differences, where * *p* < 0.05 when compared with the control (NSS) group at similar time periods and ^#^ *p* < 0.05 when compared with the treatment group at 3 h, postoperatively.

Treatment	Postoperative VAS Scores		
	3 h	4 h	5 h
NSS	44.25 ± 27.16	48.67 ± 21.43	42.63 ± 23.08
Lidocaine with epinephrine	19.44 ± 18.03 *	27.63 ± 19.26 *^,#^	39.74 ± 19.73

* Paired *t*-test, # ANOVA test under Table 2.

**Table 3:** Average amount of paracetamol consumption by volunteers at each postoperative day. Data are expressed as mean ± SD values (*n* = 28); * *p* < 0.05, ** *p* < 0.01 when compared with the treatment group at similar time periods.

Treatment	Postoperative Paracetamol Consumption (Tablet/Person)						
	Day 1	Day 2	Day 3	Day 4	Day 5	Day 6	Day 7
NSS	3.17 ± 0.78	2.27 ± 0.66	1.03 ± 0.59	0.93 ± 0.47 **	0.32 ± 0.29 *	0.09 ± 0.15	0.02 ± 0.15
Lidocaine + epinephrine	2.34 ± 0.41 **	1.81 ± 0.69 **	0.65 ± 0.49 **	0.37 ± 0.21	0	0	0

* Paired *t*-test.

## Data Availability

The original contributions presented in the study are included in the article/Supplementary Materials. Further inquiries can be directed to the corresponding author.

## Supplementary Materials

The following supporting information can be downloaded at https://www.mdpi.com/article/10.3390/dj12120412/s1: Table S1: Information of the patients who presented with bilateral mesioangular-impacted lower third molars, such as age, gender, and any medications.

## Abbreviations

ANOVA = one-way analysis of variance, CONSORT = consolidated standards of reporting trial, IANB = inferior alveolar nerve block, LSD = least significant difference, MA = mesioangular, NSS = normal saline solution, OPD = out-patient department, SD = standard deviation, SPSS = Statistical Package for the Social Sciences, VAS = visual analog scale.
