# Supplementary material for: Effectiveness of Lidocaine with Epinephrine Irrigation in Reducing Acute Pain from Surgical Removal of Mesioangular-Impacted Third Molars
Source: Dent J (Basel). 2024 Dec 17;12(12):412. doi: 10.3390/dj12120412 (PMC11674364; doi:10.3390/dj12120412)
Supplement: Supplementary file 1 [file dentistry-12-00412-s001.zip › dentistry-3084654-supplementary.pdf]

Table S1. Information of the patients who presented with bilateral mesioangular-impacted lower third molars, such as age, gender, and any medications.

Code	Gender	Age (y)	Impact low molars	Postoperative medication
MF1	M	21.5	MA	amoxicillin + paracetamol
MF2	M	21.9	MA	amoxicillin + paracetamol
MF3	M	22.6	MA	amoxicillin + paracetamol
MF4	F	20.8	MA	amoxicillin + paracetamol
MF5	F	25.0	MA	amoxicillin + paracetamol
MF6	F	20.8	MA	amoxicillin + paracetamol
MF7	F	22.9	MA	amoxicillin + paracetamol
MF8	F	20.7	MA	amoxicillin + paracetamol
MF9	F	20.9	MA	amoxicillin + paracetamol
MF10	M	20.3	MA	amoxicillin + paracetamol
MF11	F	22.6	MA	amoxicillin + paracetamol
MF12	M	22.9	MA	amoxicillin + paracetamol
MF13	M	18.3	MA	amoxicillin + paracetamol
MF14	F	20.4	MA	amoxicillin + paracetamol
MF15	F	22.7	MA	amoxicillin + paracetamol
MF16	F	20.4	MA	amoxicillin + paracetamol
MF17	M	24.6	MA	amoxicillin + paracetamol
MF18	F	19.8	MA	amoxicillin + paracetamol
MF19	F	19.9	MA	amoxicillin + paracetamol
MF20	M	23.6	MA	amoxicillin + paracetamol
MF21	M	24.2	MA	amoxicillin + paracetamol
MF22	M	23.5	MA	amoxicillin + paracetamol
MF23	F	23.6	MA	amoxicillin + paracetamol
MF24	F	21.5	MA	amoxicillin + paracetamol
MF25	F	21.7	MA	amoxicillin + paracetamol
MF26	M	20.7	MA	amoxicillin + paracetamol
MF27	M	19.8	MA	amoxicillin + paracetamol
MF28	M	21.5	MA	amoxicillin + paracetamol
	13M, 15F	21.75±1.65<br>18.3-25.0		

Abbreviations: F = female, M = male, MA = mesioangular.
